# Gastric Schwannoma: A Tumor Must Be Included in Differential Diagnoses of Gastric Submucosal Tumors

**DOI:** 10.1155/2017/9615359

**Published:** 2017-05-09

**Authors:** Bao-guang Hu, Feng-jie Wu, Jun Zhu, Xiao-mei Li, Yu-ming Li, Yan Feng, He-sheng Li

**Affiliations:** ^1^Department of Gastrointestinal Surgery, The Affiliated Hospital of Binzhou Medical University, Binzhou, Shandong, China; ^2^Centers for Disease Control and Prevention of Binzhou City, Binzhou, Shandong, China; ^3^Department of Radiology, The Affiliated Hospital of Binzhou Medical University, Binzhou, Shandong, China

## Abstract

Gastric schwannoma (GS) is a rare neoplasm of the stomach. It accounts for 0.2% of all gastric tumors and is mostly benign, slow-growing, and asymptomatic. Due to its rarity, GS is not widely recognized by clinicians, and the precise differential diagnosis between GS and other gastric submucosal tumors remains difficult preoperatively. The present study reports a case of GS misdiagnosed as gastrointestinal stromal tumor and reviews the clinical, imaging, and pathological features, treatment, and follow-up of 221 patients with GS previously reported in the English literature. Although GS is rare, the case reported in the current study highlights the importance of including GS in differential diagnoses of gastric submucosal tumors. Furthermore, the findings of the review suggest that although many cases are asymptomatic, the most common symptoms are abdominal pain or discomfort, not gastrointestinal bleeding, and malignant GSs present with clinical symptoms more commonly. Although large-sample multicenter studies on the efficacy, safety, and oncological outcomes of minimally invasive techniques are required, the findings presented herein may be helpful for clinicians when diagnosing or treating GS.

## 1. Introduction

Gastric schwannoma (GS) is a rare submucosal tumor that arises from Schwann cells in the neural plexus of the stomach. It accounts for only 0.2% of all gastric tumors, 6.3% of gastric mesenchymal tumors, and 4% of all benign tumors of the stomach [1]. GS was first described in 1988 in a study by Daimaru et al. [2], in which a series of 24 cases were examined. To date, more than 200 new cases of GS have been reported worldwide, and the findings of imaging findings and analysis of the gross features of GS have been described in some sporadic case reports and the occasional series of GS cases. However, GS is not widely recognized by clinicians, and it remains difficult to accurately distinguish GS from other gastric submucosal tumors preoperatively.

The present study reports the case of a 61-year-old woman with GS and reviews the current knowledge of GS available based on sporadic case reports and the occasional series of case reports in the literature. We hope that the findings will be useful for clinicians during the diagnosis and treatment of GS.

## 2. Case Report

**Figure 1:**
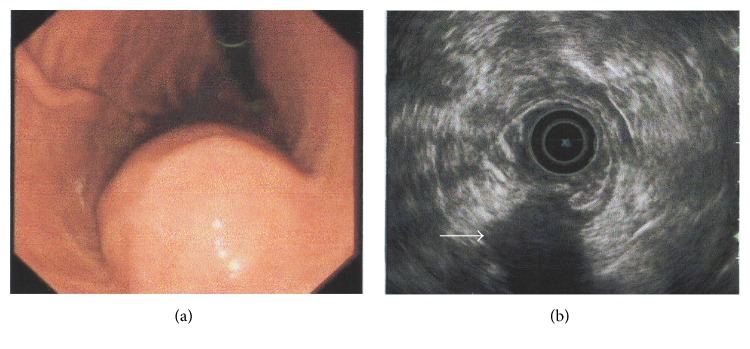
Endoscopic (a) and endosonographic (b) findings in the current case of gastric schwannoma. (a) A round, submucosal mass with an indistinct border was observed at the lesser curvature of the gastric body. (b) On endoscopic ultrasonography, the lesion (white arrow) appeared homogeneous and its echogenicity was lower than that of the normal muscle layer. The mass measured 3.7 × 3.2 cm and originated from the fourth layer.

A 61-year-old woman with a 2-year history of nonspecific epigastric abdominal pain underwent esophagogastroduodenoscopy (EGD) to rule out a digestive ulcer. EGD revealed a submucosal bulge on the anterior wall of the gastric body (Figure 1(a)). The patient then underwent endosonography, which revealed a large submucosal mass measuring 3.7 × 3.2 cm arising from the muscularis propria (the fourth layer). The features of the mass were similar to those of a gastrointestinal stromal tumor (GIST) (Figure 1(b)).

**Figure 2:**
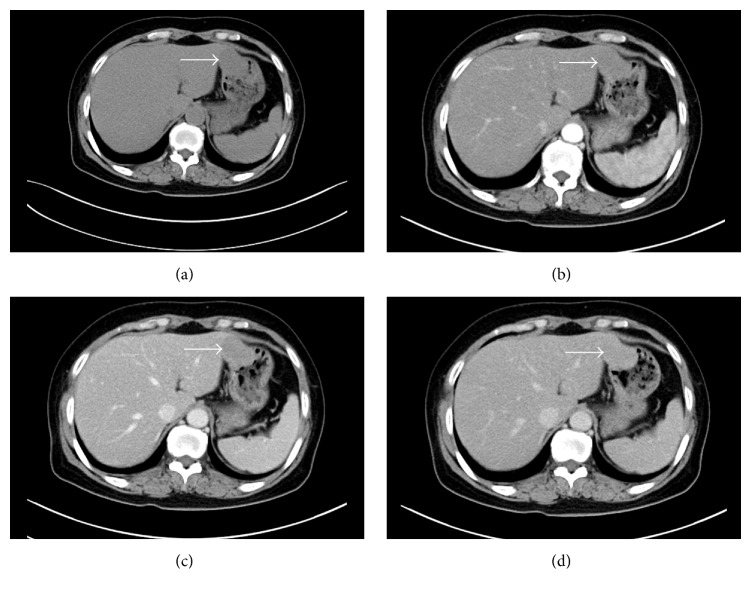
Computed tomography (CT) image of the gastric schwannoma. (a) An oval-shaped mass (white arrow) with a size of 43 × 32 mm was observed in the lesser curvature of the stomach, which exhibited a slightly low density on plain scanning CT imaging. (b) The CT value of the mass was about 38 HU before the injection of contrast medium. The mass showed delayed enhancement with a CT value of 53 HU on arterial-phase enhanced CT scanning. (c) The CT value of the mass was slightly increased (68 HU) on portal venous-phase enhanced CT imaging. (d) The CT value of the mass was increased (78 HU) on delayed enhanced CT scanning after a delay of 3 minutes.

A computed tomography (CT) scan revealed a uniformly enhancing mass located between the left lobe of the liver and the lesser curvature of the gastric body. The mass was partly exophytic and partly projected into the gastric lumen, causing smooth indentation, and measured 4.3 × 3.2 cm (Figure 2). The patient's laboratory results were unremarkable. Based on the above data, the patient was given a preoperative diagnosis of GIST arising from the anterior wall of the gastric body.

**Figure 3:**
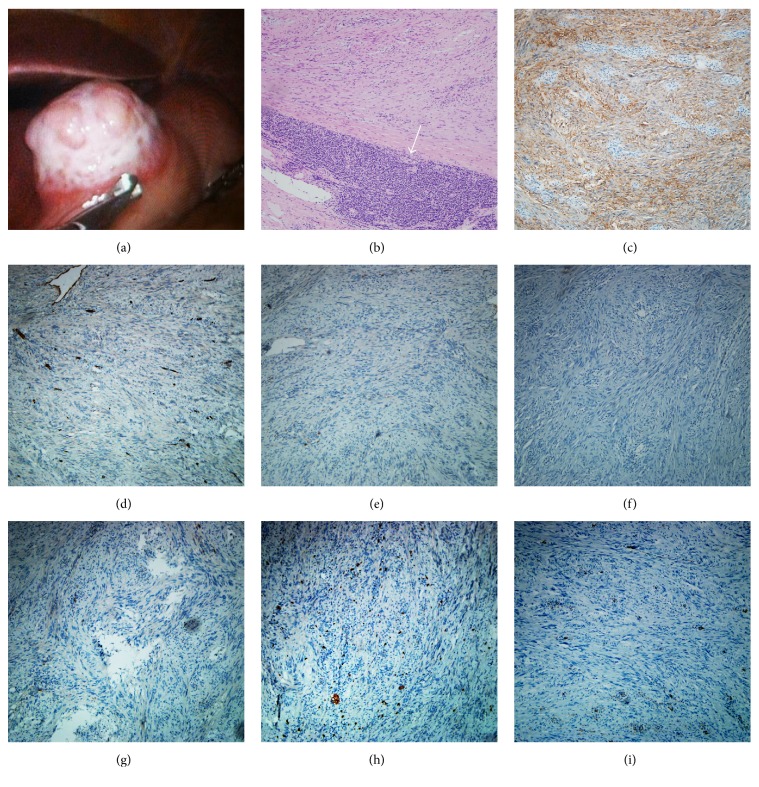
Pathological imaging of the mass. (a) An exophytic tumor with size of 4 × 3 cm arising from the anterior wall of the lesser gastric curvature was observed. The exophytic part of the mass appeared off-white in color and rough with a concavo-convex surface; however, the margin of the mass on the gastric wall was clear. (b) Hematoxylin and eosin staining showed that the mass was composed of palisade-arranged spindle cells and peritumoral cuff-like lymphocytic infiltration (white arrow). (c) Immunohistochemical staining of sections showed that the gastric schwannoma was S-100-positive but was negative for (d) CD34, (e) CD117, (f) desmin, (g) DOG1, (h) Ki-67, and (i) SMA. ×100 magnification for all micrographs.

The patient was then subjected to laparoscopic examination, which showed an exophytic tumor (4 × 3 cm size) arising from the anterior wall of the lesser gastric curvature. The exophytic part of the mass appeared off-white in color and rough, with a concavo-convex surface; however, the margin of the mass on the gastric wall was clear (Figure 3(a)). To achieve an optimal tumor-negative margin, the laparoscopy was converted to a laparotomy, and complete resection of the tumor was performed. Histopathology revealed that the tumor was composed of spindle cells in a palisading arrangement, and peritumoral cuff-like lymphocytic infiltration was also observed (Figure 3(b)). Immunohistochemical (IHC) staining showed that the spindle cells were positive for S-100 (Figure 3(c)) and negative for CD34, CD117, desmin, DOG1, Ki-67, and smooth muscle actin (SMA) (Figures 3(d)–3(i)), which confirmed a diagnosis of GS. The patient had an uneventful recovery and the 1-year follow-up examination was unremarkable.

## 3. Discussion

A review of the existing literature identified a total of 221 cases of GS (Table 1). The mean age of the patients was 56.82 ± 13.77 years (range, 10–90 years), and 191 of the 221 patients (86.43%) were aged > 40 years. Thus, it appears that GS predominantly affects adults in the fifth to eighth decades of life. The cases comprised 68 males and 153 females, with an approximate sex ratio of 1 : 2.64. Although the sex ratio in certain case series of GS has been reported as ~1 : 4 [1], we hypothesized that the gender predilection may be reduced as more cases are reported. Of the 221 reported cases, detailed clinical information was available for 164 cases. In 71 (43.3%) out of 164 cases, GS was identified incidentally, whereas 22 of the patients (11.6%) initially presented with multiple symptoms [3–21], 34 (20.7%) presented with one symptom, including abdominal pain or discomfort, and 21 cases (12.8%) were reported with gastrointestinal bleeding. These findings indicate that the majority of cases of GS are asymptomatic and that the most common initial symptom is abdominal pain or discomfort, not gastrointestinal bleeding, which differs from the findings of other case series [16]. The other symptoms, which were more rare, included palpable abdominal mass (3.05%), poor appetite (3.05%), dyspepsia (1.82%), weight loss (1.21%), and nausea or vomiting (0.6%). Recently, Yang et al. [21] reported a case of gastroduodenal intussusception due to GS, which, to the best of our knowledge, is the only case reported in English literature. In addition, we found only 1 case in which the patient initially presented with elevated serum carbohydrate antigen 19-9 preoperatively [22].

GS typically grows as a solitary lesion and is commonly located in the body of the stomach. In the current review, we found only 1 case that reported the presence of two GS lesions in the same patient [23]. The most common site of GS among all of the cases was the gastric body (59.3%), followed by the gastric antrum (26.7%) and fundus (12.0%). GS arising from the cardia was rare (2%). Additionally, the tumor size was variable: the greatest diameter size ranged from 0.8 to 15.5 cm, with a mean of 4.69 ± 2.66 cm (median: 4.0 cm).

GSs are usually benign and patients have an excellent prognosis after curative resection. Nevertheless, we identified 10 reported cases of malignant GS in the last several decades [4, 12, 24, 25], which represented 4.5% of all reported GSs. In the cases of malignant GS, 5 patients were male and 5 were females, with a mean age of 49.78 ± 22.44 years (range, 10–73 years). Among these cases, the earliest metastasis and recurrence were detected at 3 months after surgery [12]. These patients commonly presented with clinical symptoms such as abdominal pain and gastrointestinal bleeding. Thus, although this should not be considered definitive criteria by which to classify the tumors as benign or malignant, the presence of such clinical symptoms may provide valuable cues for clinicians.

The features of GS shown by imaging, including CT, magnetic resonance imaging (MRI), and [^18^F]-fluorodeoxyglucose positron emission tomography (FDG-PET), have been clearly described in several isolated case reports and some case series [26–34]. Briefly, during CT imaging, GS most commonly presented as a well-circumscribed mass with mild enhancement during the arterial phase and strengthened progressive enhancement during the venous and delayed phases. On MRI, GS typically exhibited low signal intensity on T1-weighted images and high signal intensity on T2-weighted images, which could provide further information regarding its relationship with the surrounding structures and the internal features of GS, such as signs of hemorrhage, necrosis, cystic changes, or calcification [29]. FDG-PET was usually used to evaluate the malignant potential of the lesion and to detect the recurrence or metastasis of malignant tumors [32]. Although GS, as a benign lesion, should not be FDG-avid, it was reported in certain studies that GS exhibited a relatively high accumulation of FDG during PET imaging [28, 29, 32, 35, 36] and that FDG accumulation in GS was not significantly different when compared to other submucosal lesions, such as GIST and leiomyoma [37]. Therefore, FDG-PET may be of limited value as preoperative diagnostic technique for the assessment of GS.

Endoscopic ultrasonography (EUS) is considered to be the most reliable procedure for the assessment of patients with gastrointestinal submucosal lesions [38–40], and the EUS features of GS have been systematically summarized in several case series reports [38, 41–44]. In these reports, GS commonly appeared on EUS as a round submucosal lesion arising from the fourth layer, with homogeneous internal echogenicity but without internal echogenic foci. Additionally, the echogenicity of the GS was generally lower than that of the surrounding normal muscle layers [38, 45]. Jung et al. [40] hypothesized that these findings may be helpful for differentiating GS from GIST. However, we were unfortunately unable to do so using EUS in the current described case.

Regarding its gross appearance, GS commonly presents as a yellow-white or off-white, solid, well-circumscribed, and round mural mass. Microscopic examination demonstrates that the typical cytological/morphological features of GS are palisade-arranged spindle cells and peritumoral cuff-like lymphocytic infiltration [46]. On histopathological sections, the spindle cells are predominantly located at the center of the lesion and often appear light red with hematoxylin and eosin staining in the cytoplasm. The nuclei of the spindle cells may exhibit a low degree of atypia and mitotic figures are rarely visible (<15/50 high-power fields); these are considered to be the criteria for classifying the tumor as benign or malignant [16]. On IHC sections, GSs are S-100-positive but are CD34-, CD117-, SMA-, and desmin-negative; detection of these markers is widely considered to be the gold standard for diagnosis of GS [46].

Complete surgical resection is widely considered to be a curative treatment for GS, and laparoscopic or open approaches for wedge resection, subtotal gastrectomy or near-total resection, and total gastrectomy are the treatments of choice [5, 9, 47–51]. As GS rarely metastasizes to the lymph nodes, surgical lymphadenectomy is not routinely performed and is only considered if enlarged lymph nodes are observed. Recently, minimally invasive surgical approaches, including endoscopic submucosal tunneling resection [52, 53], endoscopic enucleation [54], and endoscopic full-thickness resection with [55–57] or without [58–60] laparoscopic assistance, have been actively used as diagnostic tools and therapeutic interventions for GS. Based on short-term follow-up observations, these approaches were not associated with any severe postoperative complications. Nevertheless, to date, no large-sample multicenter studies on the efficacy, safety, and oncological outcomes of these minimally invasive surgical approaches have been published. We therefore suggest that these approaches should not be a first choice and should only be used if the diagnosis of GS is definitively confirmed.

A paper published in 2015 by Hong et al. [17] reviewed 137 cases of GS and did not identify recurrence or metastasis in any patients during a follow-up period ranging from 1 to 336 months. The authors therefore concluded that benign GS does not usually recur and, thus, frequent follow-up with CT imaging is not recommended [17]. For the current review, we retrieved 126 cases that reported detailed follow-up information, ranging from 1 to 420 months, from medical literature published in English. Recurrence and metastasis were only observed in malignant GS and not in benign cases of GS, which was similar to the results reported by Hong et al. [17]. The follow-up times in cases of malignant GS ranged from 5 to 120 months, and only 3 out of 10 patients died due to metastasis or recurrence of GS within 5 years after surgery. The earliest recurrence was detected at 3 months after surgery. In addition, Choi et al. [31] reported that the mean doubling time of GS tumors was nearly 5 years, based on CT images with a series of follow-ups. We therefore suggest that the follow-up should be conducted over a period of at least 5 years for cases of malignant GS. However, further research is necessary in order to better understand the features of malignant GS.

## 4. Conclusion

Although GS is rare, the case reported in the current study highlights the importance of including GS in differential diagnoses of gastric submucosal tumors. Furthermore, the following points regarding GS should be noted: (i) the magnitude of the gender predilection may be reduced as more cases are reported; (ii) the most common symptom is abdominal pain or discomfort, but not gastrointestinal bleeding; (iii) patients with malignant GS commonly present with some clinical symptoms; (iv) although endoscopic submucosal tunneling resection, endoscopic enucleation, and endoscopic full-thickness resection, with or without laparoscopic assistance, have been actively performed as diagnostic and therapeutic techniques for GS, large-sample multicenter studies on the efficacy, safety, and oncological outcomes are still required.

## Figures and Tables

**Table 1:** Clinical features of GS reported in English literature.

	Benign	Malignant	Overall	*P* value
Total	211	10	221	
Male/female	63/148	5/5	68/153	0.178
Average age (years)	57.13 ± 13.12	49.78 ± 22.44	56.82 ± 13.77	0.118
*Symptoms (cases)*				0.695
NA	56	1	57 (34.76%)	
Multiple symptoms	19	3	22 (13.41%)	
Asymptomatic (incidentally found)	69	2	71 (43.29%)	
Abdominal pain or discomfort	32	2	34 (20.73%)	
GI bleeding	19	2	21 (12.80%)	
Palpable mass	5	0	5 (3.05%)	
Poor appetite	5	0	5 (3.05%)	
Dyspepsia	3	0	3 (1.82%)	
Weight loss	2	0	2 (1.22%)	
Nausea or vomiting	1	0	1 (0.6%)	
*Location (cases)*				0.581
NA	83	3	86 (52.44%)	
Subcardia	2	0	2 (1.22%)	
Fundus	15	0	15 (9.15%)	
Body	82	5	87 (53.05%)	
Antrum	29	2	31 (18.90%)	
*Size (diameter, cm)*				0.897
Mean size	4.66 ± 2.62	4.66 ± 1.97	4.67 ± 2.60	
Median size	4	4	4	
*Follow-up time (months)*				0.102
Mean time	78.19 ± 84.85	44.67 ± 38.02	74.67 ± 82.59	
Median time	43	28	38.5	

NA: not available.

## Abbreviations

GS: Gastric schwannoma
GIST: Gastrointestinal stromal tumor
ESD: Endoscopic submucosal dissection
EGD: Esophagogastroduodenoscopy
CT: Computed tomography
IHC: Immunohistochemistry
MRI: Magnetic resonance imaging
FDG-PET: [^18^F]-Fluorodeoxyglucose positron emission tomography
EUS: Endoscopic ultrasonography
HPFs: High-power fields
SMA: Smooth muscle actin.
